# Deep Learning-Based Teaching Strategies of Ideological and Political Courses Under the Background of Educational Psychology

**DOI:** 10.3389/fpsyg.2021.731166

**Published:** 2021-10-22

**Authors:** Xiaoqing He, Peiyao Chen, Jieting Wu, Zhen Dong

**Affiliations:** ^1^School of Marxism, Chengdu Normal University, Chengdu, China; ^2^China Aerospace Science & Industry Corp., Beijing, China; ^3^Engineering University of Armed Police Force, Urumqi, China; ^4^School of Marxism, Sichuan Tourism University, Chengdu, China

**Keywords:** educational psychology, deep learning, Single Shot MutiBox Detector, ideological and political courses, comprehensive teaching strategy

## Abstract

At present, low teaching efficiency has been the common problem of ideological and political education in colleges and universities in China. It is essential to improve the teaching efficiency and realize the intelligent information transformation of the ideological and political courses in colleges and universities. First, the relationship between ideological and political courses and the educational psychology of college students was analyzed based on the theoretical characteristics of educational psychology and college ideological and political courses. Additionally, the teaching efficiency of ideological and political courses based on deep learning (DL) was analyzed through a literature survey. Combined with online teaching modes such as the flipped classroom and Massive Open Online Courses, a comprehensive online teaching mode of college ideological and political courses was proposed *via* educational psychology and the Single Shot MutiBox Detector networks of DL. Then, a total of 100 research subjects were selected randomly from the freshmen and sophomores of the Southwest University of Science and Technology, and their acceptability to the online ideological and political courses was analyzed by a questionnaire survey. The results show that the adopted questionnaire had high reliability and validity, and the proportion of respondents of different genders, grades, and majors was essentially balanced. More than half of the students had a good understanding of the comprehensive ideological and political courses and made progress in their values, ideology, morals, and knowledge reserves. More than half of the students had a positive attitude to the course, and they thought that the class atmosphere of the course was active, which was conducive to a satisfactory learning effect. This indicates that the teaching strategy of ideological and political courses in colleges and universities that integrates educational psychology, DL, and online information can attract students. The contribution of this study is that the research outcome can be applied to the concrete formulation of the teaching strategies of ideological and political courses for college students.

## Introduction

Ideological and political theory courses are crucial for schools to implement the fundamental task of moral education. The enhancement of the effectiveness of ideological and political courses in teaching practice has become a practical task in teaching reform. In the new era, strengthening, and improving the teaching of ideological and political courses requires a new instruction thought of education and teaching. In recent years, under the leadership of the Communist Party of China (CPC) and the government of China, the ideological and political theory teaching of colleges and universities has achieved remarkable results. In general, college students have healthy, upward, and positive beliefs, thoughts, and values (Dache et al., 2019). However, with the progress of society, ideological, and political education in colleges and universities is facing increasing challenges. Ideological and political courses are critical spiritual guidance for contemporary college students. Therefore, it is necessary to formulate effective strategies and policies to guide students in establishing a correct world outlook, values, and outlook on life (Goodson, 2020; Li and Zheng, 2021). The application of educational psychology to ideological and political teaching in college can improve the quality of teaching. The development and improvement of its theoretical system can promote the theoretical research of ideological and political teaching (Hartson et al., 2021).

Although deep learning (DL) technology has become a research hotspot in many fields, there are only a few studies on its in-depth application to classroom instruction. Deep learning that focuses on the transformation of the learning styles of students has become the cardinal direction of international education reform. Deep learning highlights the learning subjectivity of students, pays attention to the adjustment of learning methods, and emphasizes the cultivation of the paramount literacy of subjects. The implementation of DL in ideological and political courses is conducive to deepening the goal of cultivating talents, optimizing the learning methods of students, implementing the core literacy of disciplines, and promoting the new curriculum reform of ideological and political education. Teachers can implement DL in ideological and political courses by creating real situations, developing teaching topics, carrying out cooperative learning, and strengthening value guidance strategies. In the Internet era, ideological, and political courses need to be combined with modern information technologies for continuous reform and innovation. On the one hand, the issues set by teachers often lack depth and breadth or are even inferior and pseudo topics. Many issues are inadequate for the deep cooperative inquiry and discussion of students, and some students may inactively rely on other members of the group to complete the task, evading the advanced training of thinking, which reduces the effect of learning. On the other hand, lecturers cannot accurately control the activity time of issue discussions, and schedule a good chunk of time for seemingly lively discussions of the selected topic, forming prominently formalistic learning. Therefore, the current dilemma of issue-based teaching coerces the activity-based curriculum of ideological and political courses in high school into exploring more effective teaching methods. The issue-based teaching reform-oriented to DL can provide new ideas and methods for students. Hence, educational psychology was introduced into the teaching of ideological and political courses to college students. Additionally, the acceptance survey of online ideological and political courses was innovatively combined with the analysis of the state ideological and political class based on DL. This creative attempt can provide a realistic basis for the formulation of ideological and political teaching strategies for college students in the new era.

There are six sections in this study. The first section is the introduction, which describes the research background, research status, innovation points, and contributions. The second section is the literature review, which analyzes the current research background and research status of ideological and political teaching worldwide. The third section introduces the research method and analyzes the relationship between the ideological and political courses of college students and educational psychology. Moreover, an efficiency analysis model of the ideological and political class state was proposed based on the Single Shot MutiBox Detector (SSD) algorithm. A questionnaire survey on the effects and acceptance of comprehensive online ideological and political courses was performed. The fourth section expatiates the research results, focusing on the cognition of the college students of the comprehensive ideological and political courses, and formulates the teaching strategy of the comprehensive ideological and political courses. The fifth part is the discussion, which highlights the advantages of this work by comparing its research results with those of other scholars. The sixth part is the conclusion, which summarizes the research methods and outcomes and states deficiencies and prospects.

## Literature Review

### Present Situation of Ideological and Political Teaching in Colleges and Universities

Ideological and political education is primarily aimed at college students. The ideological and political courses in colleges and universities shoulder the momentous mission of cultivating qualified builders and successors of socialism, showing prominent significance. Lin (2021) stated that other countries except for China usually implicitly carry out ideological and political education through civic education, religious education, legal education, and moral education. Instant music videos have become extensively fashionable in society, and gradually became one of the sources of entertainment for contemporary college students. Song and Tian (2020) combined the short video recommendation model with the characteristics of new media to propose innovative ideas for ideological and political work in colleges and universities. Wang (2020) analyzed the artificial intelligence (AI) teaching expert system and summarized its functions and characteristics. They pointed out that the ideological and political teaching system for college students based on mobile AI terminals could be used as a teaching manager, teaching assistant, and even teaching assistant. Various countries perform ideological and political education with different concepts according to their national conditions. For instance, Frisbie et al. (2019) studied the “social studies” and “moral time” in Japan; Gupta et al. (2020) mentioned the “civic education” in the United States and France, the “life education” in Singapore, the “political education” in Britain and Canada, etc.; Yang (2021) indicated that ideological and political education in American universities serves to promote personality development, value, political education, moral education, healthy personality education, and religious education. Ford and Jennings (2020) added the Western European political profile and some social issues to the concept of higher education. Shchepetylnykova and Alvis (2020) investigated the contribution of international development activities to the overall internationalization of public higher education institutions in the United States. They concluded that international development activities promoted the mission of education, research, and service of public universities. Additionally, the ideological and political education in American universities paid attention to the combination of general patriotism and national spirit, and combined explicit courses with implicit courses, paying more attention to the educational function of implicit courses.

### Teaching Techniques of Ideological and Political Courses in Colleges and Universities

The Chinese scholar Wang et al. (2018) and Marchingiglio (2021) suggested that personality education theory should be integrated into the teaching of ideological and political courses in colleges and universities, and it was necessary to simultaneously take the personality of the students and the teaching effect into consideration. Rogoza et al. (2018) showed that factors such as human personality and self-esteem had different characteristics that influence each other, so, the idea of the coexistence of knowledge and interest and the teaching-learning transaction should be integrated into the teaching effect considering the personality of students. Moody (2020) established a data-mining model for the evaluation of teaching quality and listed its indicators. Additionally, they gave the mining process and specific classification in the analysis of association rules and determined the mining object, data selection, data-mining process, and specific mining steps in the cluster analysis. Moreover, they discussed the relationship between the basic information of teachers, teaching methods, and teaching evaluation results, generated clustering results, and finally expected the new vistas for the selection of evaluation methods, the formulation of relevant policies, and the improvement strategies of teachers' teaching.

In the Internet age, the analysis of the effectiveness of ideological and political teaching and class state through the network has become a hot topic. Nowadays, the booming DL technology has gradually been applied to study the behavior, state, and fatigue of students in classrooms (Stoeve et al., 2021). For example, Melkonian et al. (2017) and Song et al. (2021) found that the concentration degree of students in the classroom could be evaluated by profile face detection algorithms, changes in eye closure, and head-up and head-down behavior recognition. Hu and Zhang (2021) proposed an eye and nose detection method using a facial recognition model based on DL to evaluate the attention of the students in the classroom. Moreover, Wu Y. et al. (2019) proposed to identify the classroom behavior of students using Openpose bone detection. Feng (2021) expounded the importance and superiority of the “autonomy-cooperation-exploration”-type ideological and political education teaching mode in aerobics classes based on the summary of the practice of aerobics teaching. They put forward the major problems existing in the ideological and political education of aerobics teaching, and further optimized aerobics teaching through ideological and political education, to effectively improve the specific teaching effect. Samfira and Sava (2021) believed that the teachers who adopted a custodial view on the pupil control ideology endorsed more dysfunctional beliefs than the teachers who adopted a humanistic view. They found that they tended to present a higher level of perfectionism, unrelenting standards, and problematic relational beliefs, including schemas of mistrust and entitlement, and more often, they also presented other-directed demands and derogation of other thoughts. Such results described the view on the dysfunction of students with misconduct, who oppose their strict and/or perfectionist expectations. In summary, all countries attach great importance to the ideological and political education of college students. The effective education methods of the implementation of ideological and political education of college students have raised considerable concern from the present academia. In this study, fundamental research is performed on teaching technology and the current situation of ideological and political education.

### Innovation and Contribution

In the field of ideological and political education, many scholars have conducted abundant research. In this innovation report, the relationship between the ideological and political courses of college students and educational psychology was analyzed, and a state efficiency analysis model of the ideological and political class was proposed based on the SSD algorithm. Moreover, a feedback survey was performed on the effect of the network ideological and political course.

## Methods

### The Relationship Between the Ideological and Political Courses of College Students and Educational Psychology

**Figure 1:**
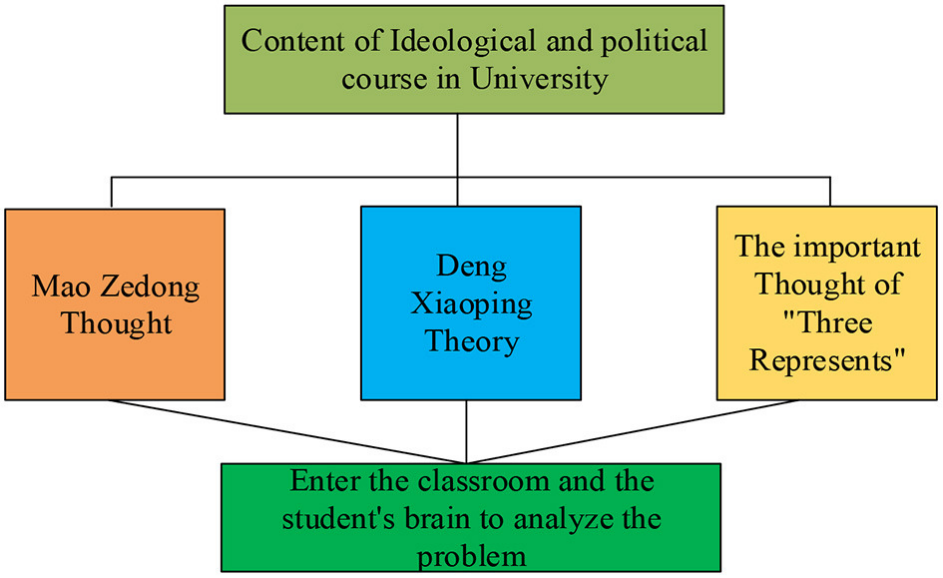
Elementary contents of the ideological and political course.

The core content of ideological and political education is the theoretical practice of Marxism in China, namely, Mao Zedong Thought, Deng Xiaoping Theory, and the important thought of “Three Represents” (Li and Guan, 2020), as shown in Figure 1.

The elementary contents of the ideological and political course should rely on the systematic education of Marxism–Leninism, Mao Zedong Thought, Deng Xiaoping Theory, and the important thought of “Three Represents.” It is necessary to dexterously introduce the Deng Xiaoping Theory and the important thought of “Three Represents” into textbooks, classrooms, and college courses. The course should also help college students in learning and mastering the basic viewpoints and scientific system of the socialist theory with Chinese characteristics and guide college students to use the Marxist world outlook and methodology when understanding and analyzing problems. It is essential to guide students to develop Marxist educational thought, life values, morals, and the legal system. In addition, ideological and political educators should guide students to cultivate lofty ideals and good moral qualities and to exhibit the excellent tradition and spirit of the times of the Chinese nation according to value standards and behavior norms. Moreover, the education in modern Chinese history can help students in understanding national history, national conditions, and the reason for the choice of Marxism, the CPC, and the socialist road. It is necessary to carry out the education of the line, guidelines, and policy of the CPC to enable students to correctly understand the international situation and the necessity to accept the ideological and political courses based on the theory of educational psychology (Golovin and Vissonov, 2021). The psychological characteristics formed at school will also greatly affect the subsequent careers of students. Wu W. et al. (2019) showed that personal psychological characteristics had a strong impact on entrepreneurial intention under the intermediary effect of self-efficacy.

As the main approach of ideological and political education for college students, ideological, and political courses can not only promote the development of universities and the coordinated development of students from all aspects but also is of vitally strategic significance to the cultivation of socialist builders and successors. Table 1 illustrates the multi-dimensional teaching goals (Renshaw and Bolognino, 2016) from different perspectives.

**Table 1:** Teaching goals of college ideological and political courses from different perspectives.

**Perspective**	**Teaching goal**
Entirety	Strive to cultivate ideal, cultural, moral, and disciplined builders and successors who committed to the cause of socialism with Chinese characteristics.
Society	Give full play to the role of ideological and political education in improving students' ideological and moral quality, make students become the talents needed by society, cultivate qualified citizens, and promote social development.
Individual	Scientifically guide students to establish a correct world outlook, outlook on life, values, and guide the development direction of students, to realize the teaching goal of promoting the harmonious development of students' body and mind.
Teaching content	Include socialist thought and theory education, professional ethics education, patriotism and collectivism education, legal education, excellent traditional education, aesthetic education, and other aspects of teaching objectives.

The research object of educational psychology (Greenbaum et al., 2016) is “the psychological activity of teachers and students in the teaching process and the law of psychological activity under the background of school education, and the law of psychological activity during teacher-student interaction.” Therefore, educational psychology is a science that studies the learning situation of students, the teaching situation of teachers, and the psychological activities and corresponding laws in the process of interaction (Turner and Nolen, 2015). The influence of educational psychology on students extends to the adaptability of students to work stress in later periods. Chen (2019) proved that work stress and work engagement in high-tech industries were affected by the adaptability of the employees to enterprise culture and psychological pressure. Therefore, it is crucial for students to cultivate their psychological ability in their student period.

The relationship between ideological and political courses and educational psychology is primarily manifested in three aspects. First, they both have a common educational object, namely, students. Second, they both include content such as belief and will. The firm ideals, beliefs, and perseverance of students will not only improve their moral level but also help them to form positive and healthy psychological qualities. Third, they have a common goal of talent cultivation. Overall, the goal of ideological and political education is to “cultivate ideal, moral, cultural, and disciplined builders and successors dedicated to the cause of socialism with Chinese characteristics” (Metaferia et al., 2021). Educational psychology aims to investigate the mental health of students through their learning of skills and cultural knowledge, ideology and morals, and behaviors and habits to promote the integrated quality of students.

### Analysis of the Teaching Efficiency of Ideological and Political Courses Based on DL

In a traditional classroom, teachers and schools need to keep abreast of the class state during the class through manual monitoring to judge the learning efficiency, acceptance, and attendance of students. With the development of AI and smart campuses, the application of intelligent technologies to conventional class sessions by transforming manual monitoring into intelligent recognition has become a research trend and hotspot (Reyes et al., 2019).

**Figure 2:**
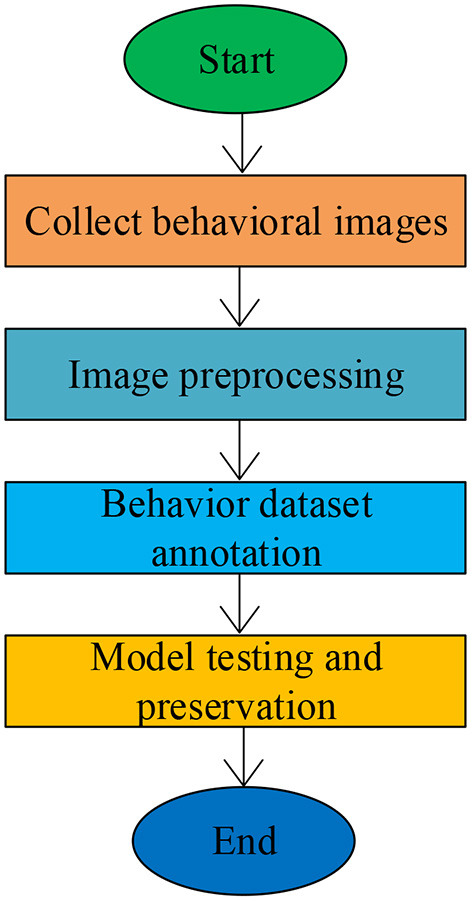
The process of student behavior recognition.

This section aims to analyze the recognition efficiency of students in class by relying on the DL model (Xing et al., 2021). Based on the literature review, the SSD algorithm (Pan et al., 2021) is used to identify five common behaviors of students in the classroom, namely, sitting, listening, writing, sleeping, raising hands, and playing with mobile phones. Figure 2 reveals the procedure of student behavior recognition.

First, it was necessary to collect as many images of the behavior of students in classrooms as possible of the five student behaviors, namely, raising hands, sitting, writing, sleeping, and playing with mobile phones. Second, a behavior recognition database was established. After preprocessing and labeling, the collected images were divided into three parts, namely, a training set, a test set, and a verification set (Wang et al., 2019). Finally, the model was trained and tested. The training set was put into the behavior recognition model for the initial training of the model, and then the validation set was utilized to verify this model. The parameters of the behavior recognition model were adjusted according to the validation results.

The test set was ultimately input into the model for the results, which were then analyzed to determine whether they were identical to the expectations. The comparison result decided the model for the next training. The behavior recognition model with a good recognition effect was saved for the subsequent recognition experiment on the behavior of students.

### Survey on Acceptance of the Comprehensive Online Ideological and Political Course of College Students

(1) The ideological and political courses were conducted using online teaching modes such as the flipped classroom and Massive Open Online Courses (MOOC) (Sharp et al., 2020). Meanwhile, DL was used to analyze the states of the students in class, which is in line with the concept of information-based teaching in the new era. Wu and Song (2019) stated that the adaption of media in entrepreneurship courses should fully consider the psychological acceptance of college students. The acceptance of students of network ideological and political education refers to the process whereby college students actively select and integrate the needed ideas, political views, and moral norms required by the education on the network based on their ideas and values so that t quality and practical behavior guidance can be formed.

(2) A questionnaire was designed for college students to investigate their acceptance of online ideological and political courses. The questionnaire was mainly divided into two parts. The first part is the basic information, including gender, grade, major, time online (Question 1 to Question 4). The second part is the investigation of the cognition and acceptance of online ideological and political education (Question 5 to Question 8) and acceptance elements (Question 9 to Question 21). The survey on the acceptance elements involve four aspects: (1) the evaluation of the accepted contents of online ideological and political courses of college students; (2) the evaluation of their acceptance ability; (3) the evaluation of online ideological and political teachers; (4) the evaluation of the acceptance carrier of the online ideological and political course of college students. Therefore, the questionnaire tackles the current situation and problems of the acceptance of college students of online ideological and political courses, followed by the reasons for these problems. Moreover, there is an open subjective question (Question 22) at the end of the questionnaire to make the research more comprehensive and rigorous. Table 2 describes the content of the questionnaire.

**Table 2:** Online ideological and political course acceptance questionnaire.

Q1: Your gender	A: Male B: Female
Q2: Your grade	A: Freshman B: Sophomore C: Junior D: Senior
Q3: The subject category of your major	A: humanities and social Sciences (Philosophy, History, Literature, Education, Law, Economics, Management) B: natural sciences (Science, Engineering, Agriculture, Medicine)
Q4: The amount of time you spend on the Internet	A: 1–3 h B: 3–6 h C: More than 6 h
Q5: Your recognition to the ideological and political education work of the school	A: recognized B: slightly recognized C: not recognized
Q6: Who do you think is the main body of ideological and political education in school?	A: instructors B: ideological and political course teachers C: teachers of other majors
Q7: Do you think ideological and political courses in university have practical significance?	A: greatly significant B: significant C: slightly significant D: not significant
Q8: What is the reason for you to study ideological and political courses?	A: for life instruction B: compulsory reason C: personal interest
Q9: In what aspect do you think the ideological and political courses have improved you the most?	A: political events B: moral behavior C: thinking pattern D: no improvement
Q10: What do you hope to achieve through ideological and political education?	A: knowledge and skills B: volitional quality C: mode of thinking D: comprehensive quality
Q11: The positive factor inducing you to take ideological and political courses are	A: teachers' high knowledge level and teaching ability B: practically significant contents C: interest in contents related to ideology and politics D: a certain understanding and foundation of ideological and political education
Q12: The negative factor hindering you from ideological and political courses	A: poor social environment B: dislike for the teaching method of the teacher C: lacking interest in ideological and political content D: unpractical contents
Q13: What problems do you think exist in ideological and political courses?	A: The content is too difficult. B: The textbook is difficult to understand. C: The teaching method is not interesting. D: The students do not care. E: The course does not reflect the practical significance. F: Others.
Q14: What do you think of the content of the politics course?	A: very valuable B: out of date C: of no practical use
Q15: What qualities do you think the ideological and political teachers you have come into contact with lack?	A: sense of humor B: ability to interpret the textbook in a simple way C: enthusiasm for work D: profound knowledge E: ability to adjust the class atmosphere F: concern for students
Q16: What kind of teaching content do you prefer in ideological and political courses?	A: pure theory with knowledge points B: theories followed by cases C: full of cases like stories D: others
Q17: Which teaching method do you prefer in ideological and political courses?	A: traditional teaching B: teacher-student interaction, and discussion learning C: independent learning with instruction from teachers D: extracurricular study with social practice
Q18: Do you like to actively use mobile Internet terminal devices (such as mobile phones and computers) to watch cultural and ideological videos involving thoughts, politics, and philosophy worldwide?	A: extremely dislike B: dislike C: slightly like D: like E: extremely like
Q19: Your mental condition:	A: optimistic, cheerful, and positive B: mildly depressed C: negative and pessimistic, with a lot of pressure
Q20: The person you want to vent your stress to:	A: teachers B: friends C: parents D: lover E: network
Q21: Your personal value depends on:	A: social contribution B: wealth C: respect from others D: power E: knowledge or ability F: comfort G: social status H: others
Q22: What do you think needs to be improved in school network ideological and political education?	

(3) The research subjects were randomly selected from the freshmen and sophomores at the Southwest University of Science and Technology. A total of 100 questionnaires were distributed, and 93 valid questionnaires were collected, with a recovery rate of 93%.

(4) The IBM SPSS 26.0 software (IBM, Armonk, New York, United States) was used to process the questionnaire data, and Origin 201864Bit (OriginLab, Northampton, Massachusetts, United States) was used to visualize the data.

## Results

### The Reliability Test and Validity Test of the Questionnaire

**Figure 3:**
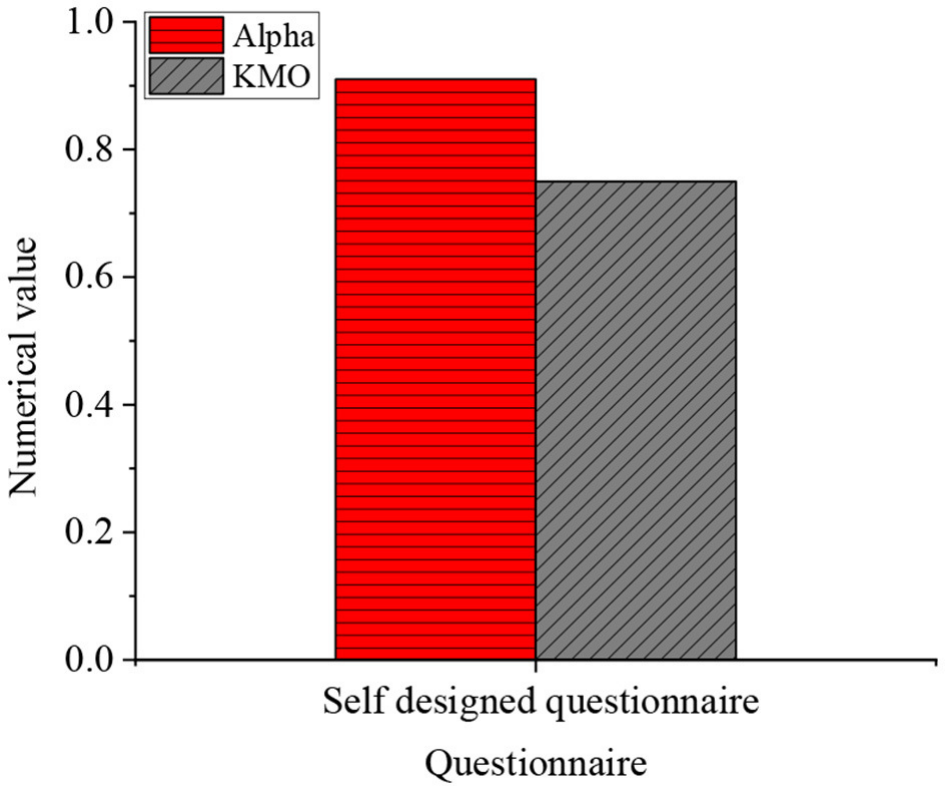
Results of the reliability and validity of the questionnaire.

Figure 3 provides the results of the reliability and validity of the questionnaire tested by IBM SPSS 26.0.

In Figure 3, the alpha coefficient of the questionnaire is more than 0.9, indicating that the reliability of the questionnaire is brilliant. The Kaiser–Meyer–Olkin (KMO) statistics are more than 0.7, demonstrating that the questionnaire has excellent validity and meets the research standard.

### Analysis Results of the Basic Information of the Research Subjects

**Figure 4:**
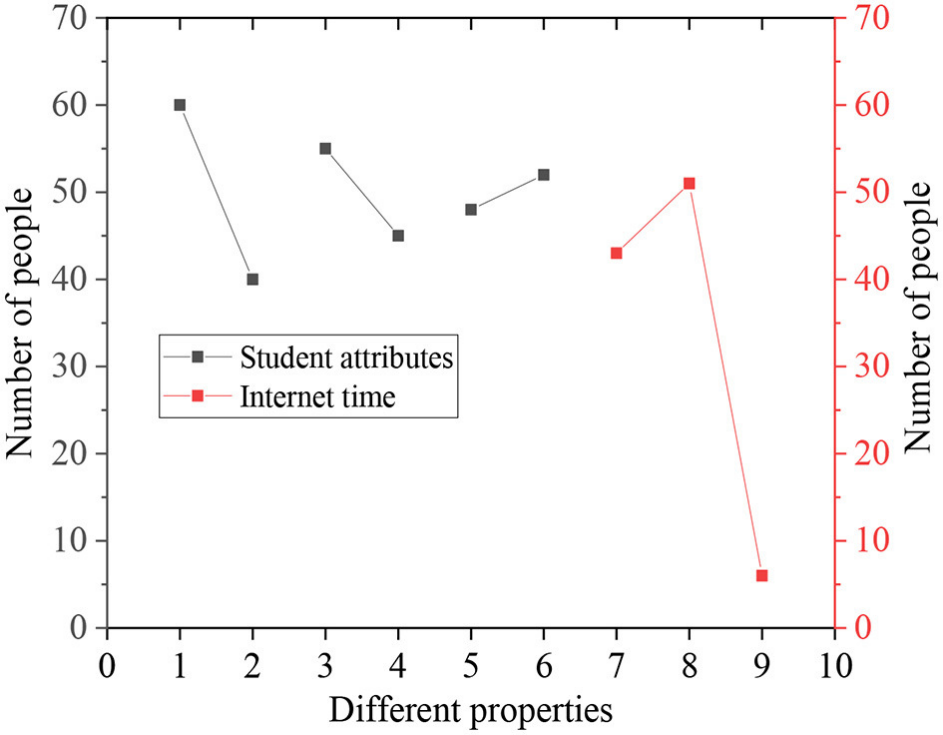
Statistical results of basic information of the research object (1: male; 2: female; 3: freshman; 4: sophomore; 5: liberal arts students; 6: science students; 7: time online < a year; 8: time online: 1–3 years; 9: time online >3 years).

Figure 4 shows the data of the basic information of the 100 research subjects, including gender, grade, major, and time online.

In Figure 4, among the randomly selected research subjects, there were 60 men, 40 women, 55 freshmen, 45 sophomores, 48 liberal arts students, and 52 science students. There were 43 students who started to use the network within a year, 51 students had access to the Internet for 1–3 years, and only six students surfed on the Internet for more than 3 years. This demonstrates that the number of male subjects is slightly larger than that of female subjects, and the number of freshmen is slightly larger than that of sophomores.

### The Cognition and Acceptance of Comprehensive Ideological and Political Courses of College Students

**Figure 5:**
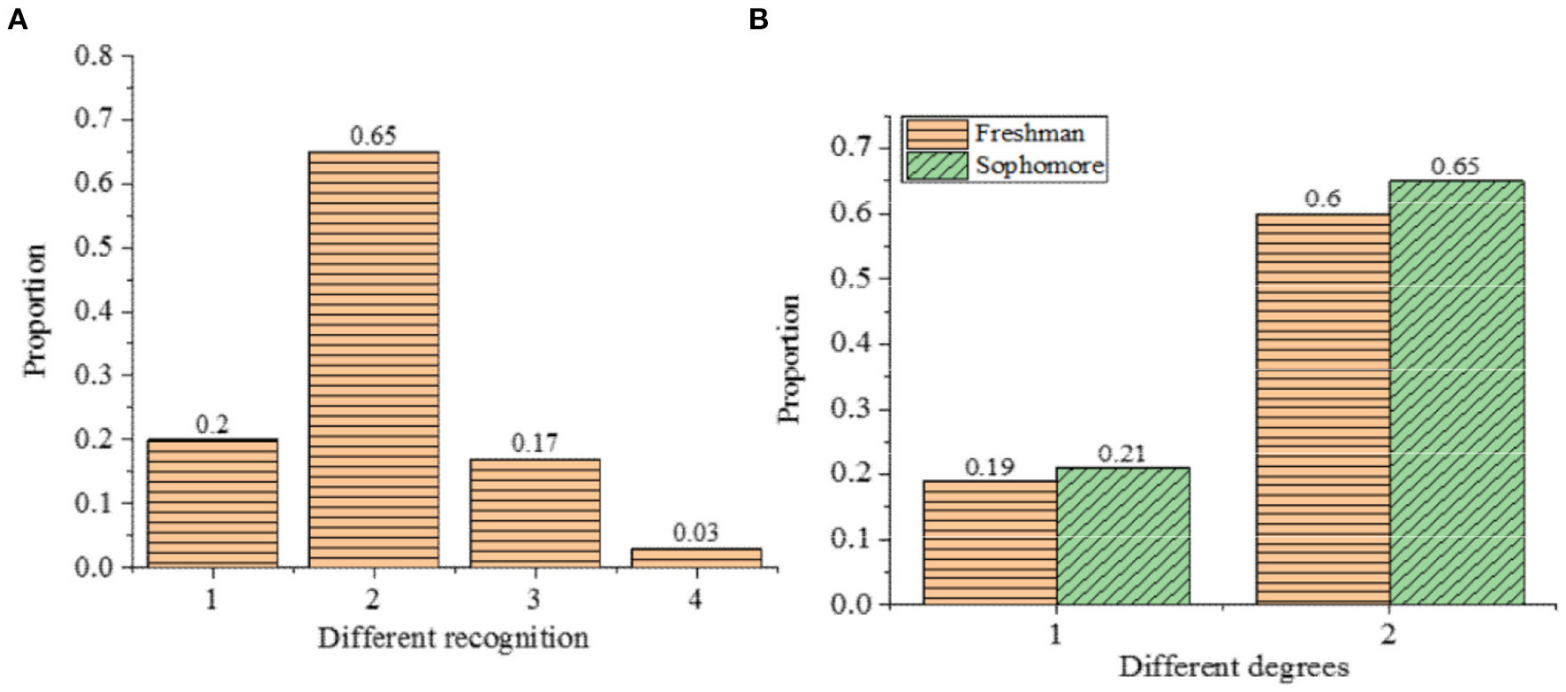
The cognition of comprehensive ideological and political courses of college students (**A**: overall awareness; **B**: gap analysis; 1: know a lot; 2: partly know; 3: heard of 4: never heard of).

Figure 5 displays the survey results of the cognition of comprehensive ideological and political courses of college students.

Figure 5 shows that 20% of the research subjects know a lot about the comprehensive ideological and political courses. The majority of students know a part of the course, amounting to 65% of the research subjects. There were 17% of the research subjects who have heard of the courses, while 3% have never heard of it. Between freshmen and sophomores, there is a small gap in the proportion of students who know a lot about the comprehensive ideological and political courses and those who partly know, which differed by 2 and 5%, respectively. This result proves that the current college students, especially freshmen, do not have a deep understanding of the ideological and political courses. Therefore, it is necessary to generate effective teaching strategies to make college students have a comprehensive understanding of the ideological and political courses.

### Influencing Factors and Learning Effects of the Learning Psychology of College Students

**Figure 6:**
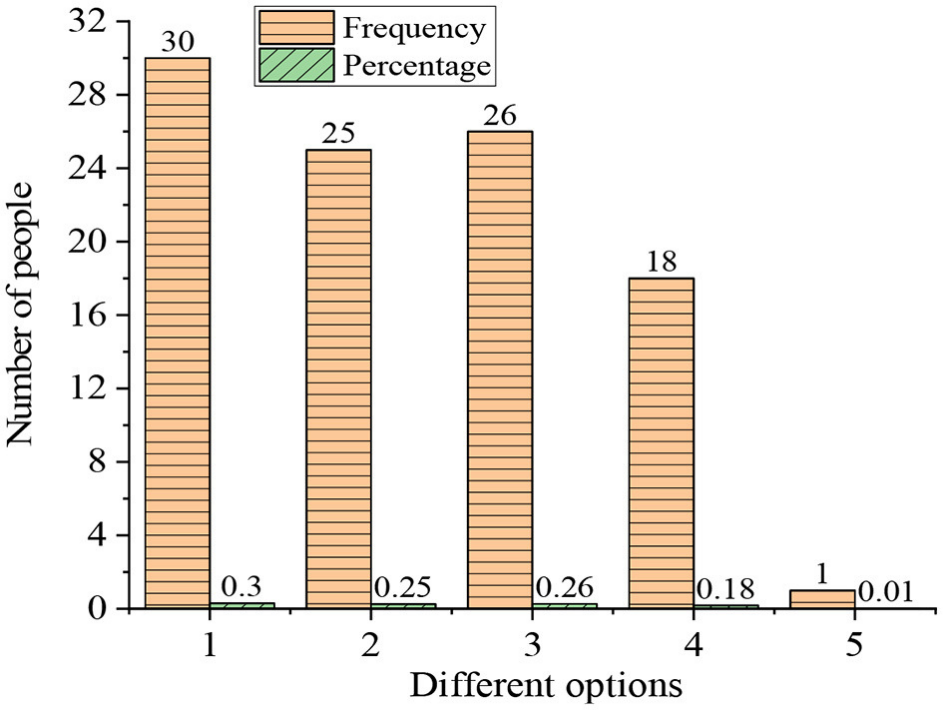
Statistical results of the gain of college students from the ideological and political courses (1: correct “three outlooks”; 2: improvement of the ideological and theoretical level; 3: improvement of moral cultivation; 4: knowledge reserves; 5: almost no gains).

The evaluation of students of their gains from the ideological and political courses is an elementary indicator of teaching efficiency. Figure 6 signifies the gains of the research subjects from the systematic learning of the ideological and political course.

According to Figure 6, most of the research objects have made great achievements after the comprehensive ideological and political courses. There were 30% of the students establishing a correct world outlook, outlook on life, and values, with 25% improving their ideological and theoretical level, 26% improving their moral cultivation, and 18% enhancing their knowledge reserves. There was only 1% of the students who have little gain from the ideological and political courses.

**Figure 7:**
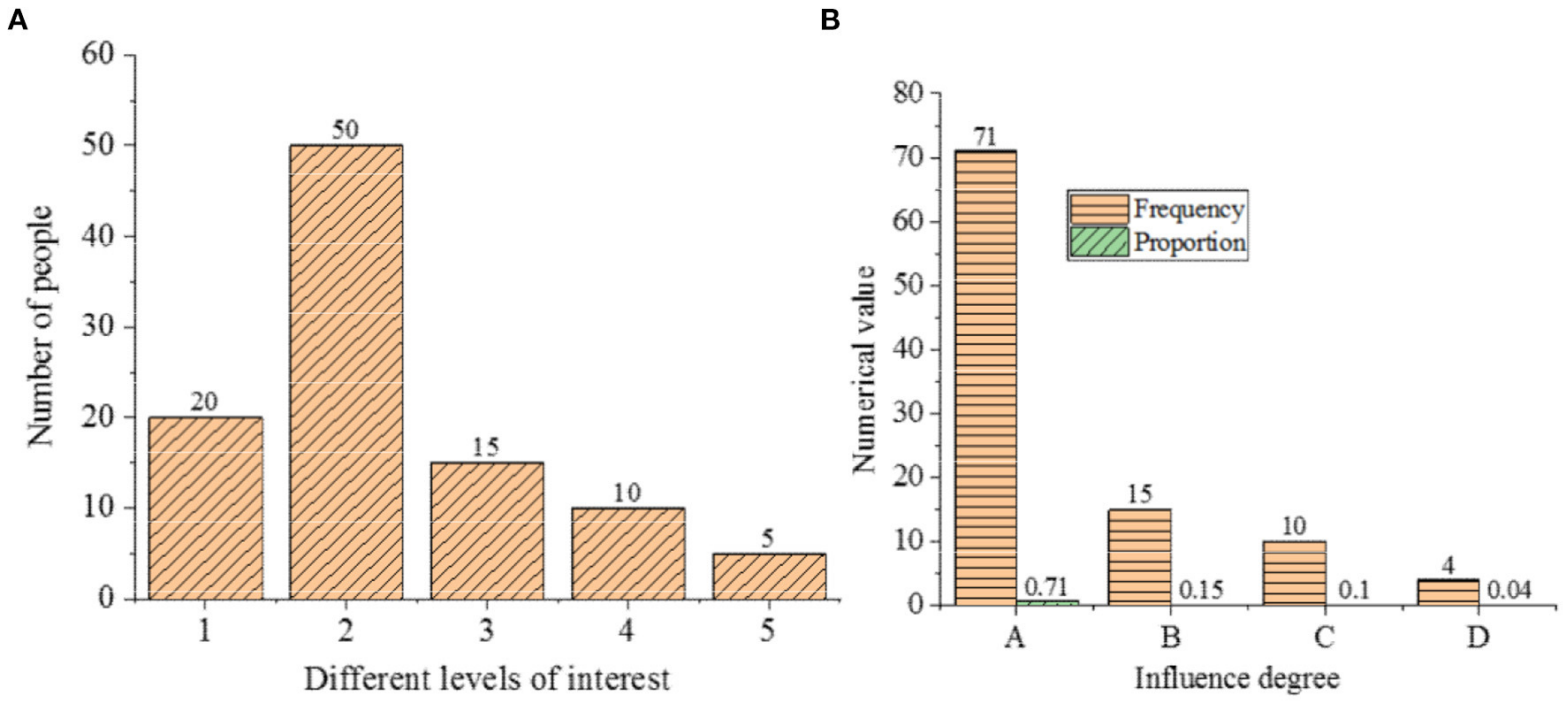
Influence of the interests and attitudes of students on the learning effect (**A**: degree of interest; 1: very interested; 2: relatively interested; 3: not very interested; 4: **A** little disgusted; 5: very disgusted; **B**: influence degree; A: greatly B: generally; C: partially; D: little).

Figure 7 shows the influence of the interests and attitudes of students on the learning effect of the ideological and political courses.

According to Figure 7, 20% of the research objects are very interested in ideological and political courses, along with 50% who are relatively interested, 15% not very interested, 10% a little disgusted, and 5% very disgusted. Furthermore, 71% of the students think that learning attitude has a great influence on the learning effect of the ideological and political courses, and 15% think that the influence is general. Additionally, 10% think that the influence is small, and only 4% think that attitude has little influence on the learning effect of the ideological and political course. This proves that it is very important to cultivate the attitude of students to the study of ideological and political courses, which will affect the learning effect of such courses.

**Figure 8:**
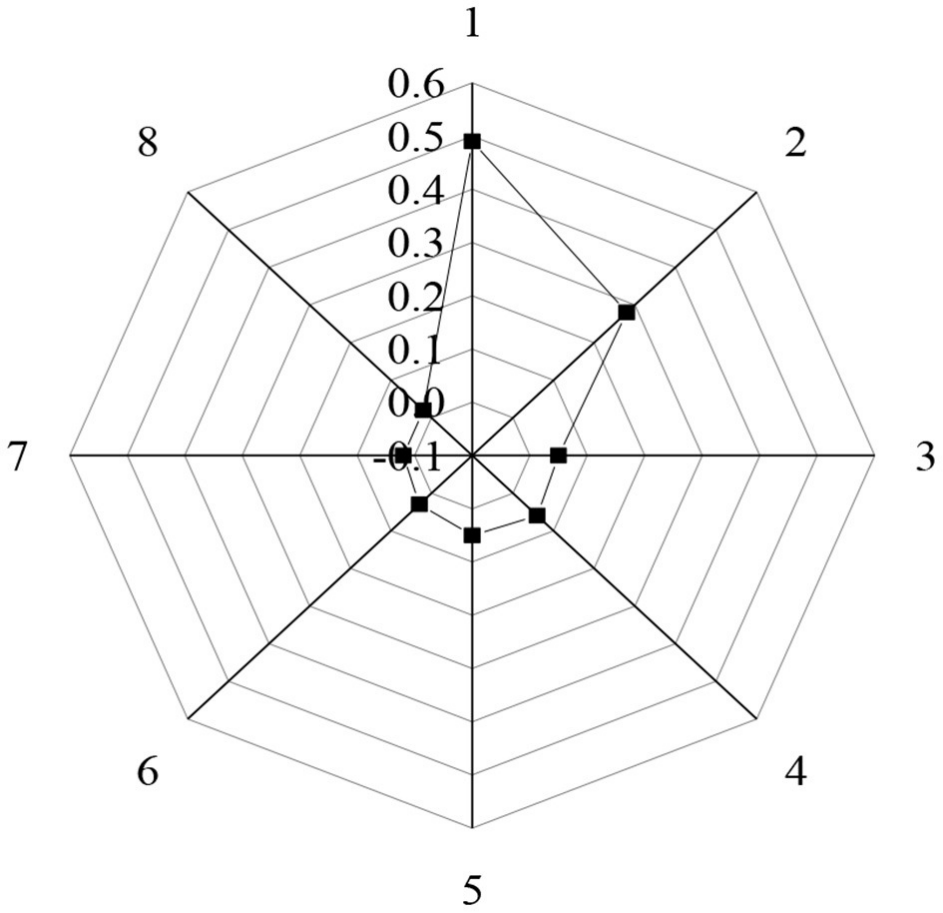
The learning motivation of students to ideological and political courses (1: compulsory course; 2: test credits; 3: discipline constraints; 4: interest; 5: teachers' personality charm; 6: teachers' vivid lectures; 7: further study; 8: enhance the theoretical basis).

In addition to learning interest and learning attitudes, the influencing factors of the learning effect on students contain learning motivation as shown in Figure 8.

In Figure 8, 49% of the research subjects took ideological and political courses as compulsory courses. Furthermore, 28% of the students attend the ideological and political courses for credits, and 5% are forced by the discipline. Additionally, 6% of the students took ideological and political courses due to interest, 5% due to the personality charm of the teacher, and 3% due to the vivid lectures of the teacher. Of the students, 2% also have a requirement for further study, and another 2% to enhance the theoretical basis.

**Figure 9:**
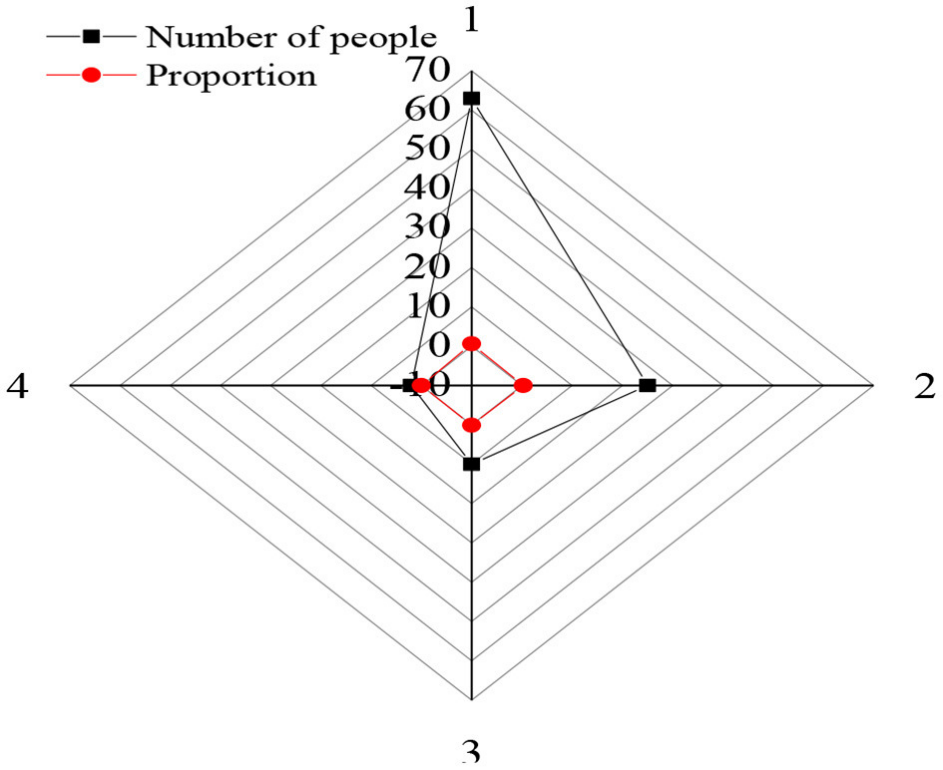
Statistical results of the class atmosphere (1: active; 2: boring; 3: dull; 4: depressed).

Figure 9 represents the statistical results of the class atmosphere of the ideological and political courses.

Figure 9 shows that 63% of the students think that the class atmosphere is active, while 25% think that it is boring, with 10% thinking dull, and 2% thinking depressed. This shows that although more than half of the students think that the atmosphere of the ideological and political courses is active, nearly 40% of the students still think that the course is boring and its atmosphere is dull. Therefore, the teaching methods of this course also need to be improved.

### Teaching Strategies of Comprehensive Ideological and Political Courses for College Students

**Figure 10:**
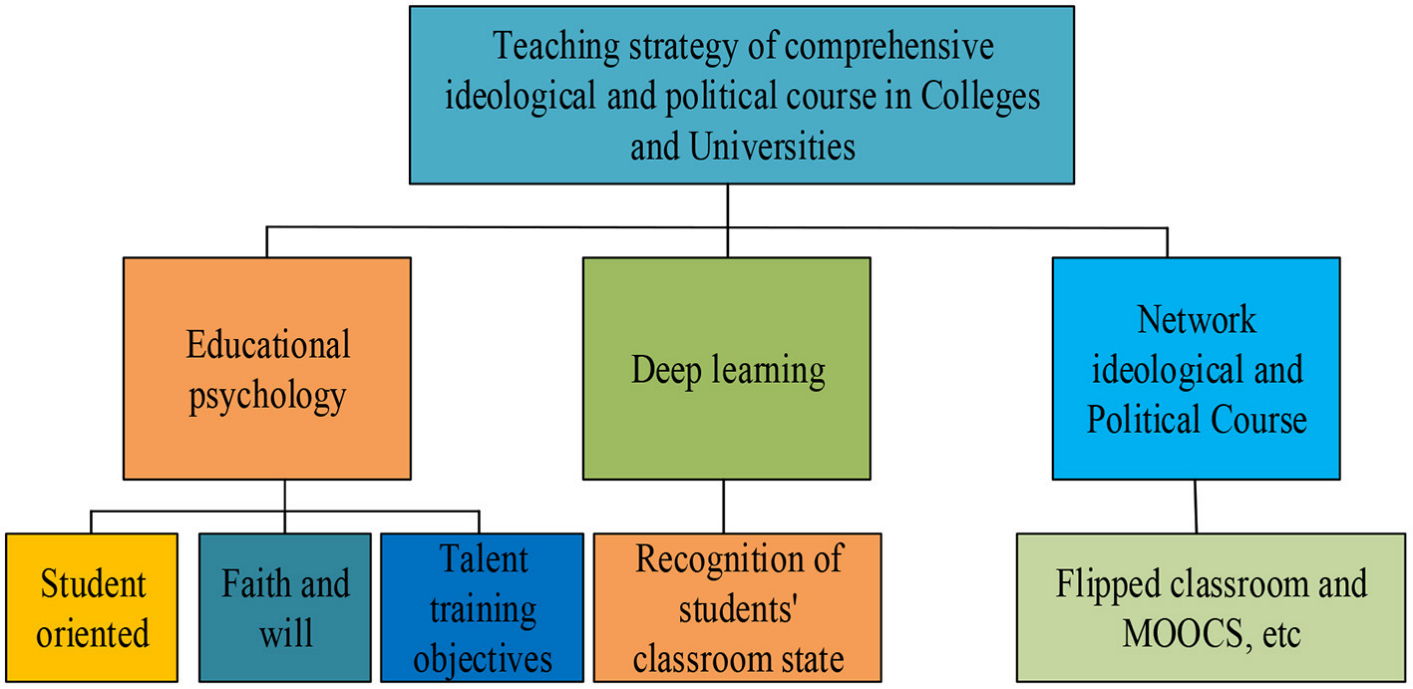
Teaching strategies of comprehensive ideological and political courses for college students.

A comprehensive teaching strategy of ideological and political courses in colleges and universities is formulated through the integration of educational psychology with the behavior recognition model of the students in the class based on DL and online ideological and political courses, as shown in Figure 10.

From Figure 10, the teaching strategy proposed for the comprehensive ideological and political courses contains the integration of educational psychology, DL, and online ideological and political course teaching. The integration of educational psychology and college ideological and political courses should regard the students as the main body, enhance the beliefs and will of these students, and cultivate builders and successors with ideals, morality, culture, and discipline dedicated to the cause of socialism with Chinese characteristics. Deep learning is mainly used to identify the class status of students, combined with flipped classrooms and online teaching such as MOOC, to truly realize the informatization and intelligent transformation of the ideological and political education strategy in colleges and universities.

## Discussion

The teaching of ideological and political courses is a process of spiritual communication, cultivating the positive learning attitudes, and interests of students through education and satisfying the psychological needs of educators and students (Gao and Liu, 2021; Zhang and Sun, 2021). Glveanu (2020) proved that attention should be paid to the rhythm and nature of psychological development to prevent the ineffectiveness of education, and it is essential to combine education with psychology, which is consistent with the results of this study. The ideological and political education in colleges and universities should critically employ the psychological effect of education, take students as the main body to play their positive roles in the course, overcome the adverse influences, and optimize the teaching concept of teachers.

As the recipients of information-based ideological and political education, college students of different grades and majors have different levels of awareness regarding online ideological and political education. Insufficient knowledge storage and comprehension of course content are crucial influencing factors of the learning effect (Ai, 2021; Bai and Zhou, 2021). Hossain et al. (2019) showed that, from the perspective of educators, the targeted online ideological and political activities should be carried out based on the concrete needs and psychological characteristics of college students. This is consistent with the research results reported in this study. For the information-based ideological and political education of college students, it is necessary to consider the characteristics of students of different genders, grades, and majors and dynamically analyze the classroom status of students and the online teaching mode. Additionally, a suitable and comprehensive teaching strategy is required for the ideological and political courses of college students, which is also important for the improvement of the performance of college students in their later careers. Qian et al. (2018) also stated that empowerment activities should be combined with task performances and psychological ideas to obtain a series of feedback.

In summary, it is necessary for the teaching strategy of ideological and political courses in colleges and universities to grasp the background of information development in the new era and conform to the frontier of science and technology with practical application values. Only in this way can the teaching efficiency of ideological and political courses in colleges and universities be truly improved to change the dull class atmosphere of ideological and political courses.

## Conclusion

This research focuses on the theoretical basis of educational psychology and its relationship with ideological and political courses in colleges and universities. Through a literature analysis, the SSD algorithm was used for the recognition of the class status of college students regarding the ideological and political courses in colleges and universities. Additionally, a comprehensive teaching strategy of information-based ideological and political courses for college students was proposed, inspired by online teaching methods such as the flipped classroom and MOOC. The research results show that students have made great achievements in the establishment of “three views,” the improvement of ideological and moral quality, and the increase of knowledge reserves after the course. Half of the research subjects were more familiar with and interested in the comprehensive ideological and political courses. Moreover, more than half of the students thought that the class atmosphere of the comprehensive ideological and political courses is active. The integration of educational psychology and ideological and political courses in colleges and universities should take students as the main body and enhance the beliefs and will of these students. The application of DL chiefly aims to identify the class status of the students and realize the intelligent information transformation of educational strategies of ideological and political courses in colleges and universities *via* the flipped classroom and MOOC.

## Data Availability Statement

The raw data supporting the conclusions of this article will be made available by the authors, without undue reservation.

## Ethics Statement

The studies involving human participants were reviewed and approved by Sichuan Tourism University Ethics Committee. The patients/participants provided their written informed consent to participate in this study. Written informed consent was obtained from the individual(s) for the publication of any potentially identifiable images or data included in this article.

## Author Contributions

All authors listed have made a substantial, direct and intellectual contribution to the work, and approved it for publication.

## Funding

This work was supported by Humanities and Social Sciences projects of the Ministry of Education (18YJA760020); Periodical achievement of 2020 Research on the Living Inheritance of Chinese Excellent Traditional Culture and Innovation of Ideological and Political Education by Chengdu Normal University.

## Conflict of Interest

PC was employed by the China Aerospace Science & Industry Corp. The remaining authors declare that the research was conducted in the absence of any commercial or financial relationships that could be construed as a potential conflict of interest.

## Publisher's Note

All claims expressed in this article are solely those of the authors and do not necessarily represent those of their affiliated organizations, or those of the publisher, the editors and the reviewers. Any product that may be evaluated in this article, or claim that may be made by its manufacturer, is not guaranteed or endorsed by the publisher.

## References

[B1] Ai H. (2021). The influence of new media on college students' ideological and political education and the countermeasures. J. Contemp. Educ. Res. 5, 44–46. 10.26689/jcer.v5i5.2125

[B2] Bai Y. Zhou X. (2021). Research on the innovation of College Counselors' Ideological and political work methods based on computer aided technology. J. Phys. Conference Series 1744:042102. 10.1088/1742-6596/1744/4/042102

[B3] Chen M. (2019). The impact of expatriates' cross-cultural adjustment on work stress and job involvement in the high-tech industry. Front. Psychol. 10:2228. 10.3389/fpsyg.2019.02228 31649581 PMC6794360

[B4] Dache A. Haywood J. M. Mislán C. (2019). A badge of honor not shame: an afrolatina theory of black-imiento for U.S higher education research. J. Negro Educ. 88, 130–145. 10.7709/jnegroeducation.88.2.0130

[B5] Feng S. (2021). Practice of “Independent-Cooperative-Inquiry” based ideological and political education teaching model in aerobics classes at colleges and universities, in 2020 12th International Conference on Measuring Technology and Mechatronics Automation (ICMTMA) (IEEE), 914–917. 10.1109/ICMTMA50254.2020.00197

[B6] Ford R. Jennings W. (2020). The changing cleavage politics of Western Europe. Ann. Rev. Politic. Sci. 23, 1–20. 10.1146/annurev-polisci-052217-104957

[B7] Frisbie S. H. Mitchell E. J. Roudeau S. Domart F. Carmona A. Ortega R.. (2019). Manganese levels in infant formula and young child nutritional beverages in the United States and France: comparison to breast milk and regulations. PLoS ONE 14:e0223636. 10.1371/journal.pone.0223636 31689314 PMC6830775

[B8] Gao J. Liu H. (2021). Problems existing in the network practice teaching of university ideological and political theory course in the context of internet. J. Phys. 1852:042075. 10.1088/1742-6596/1852/4/042075

[B9] Glveanu P. V. (2020). A sociocultural theory of creativity: bridging the social, the material, the psychological. Rev. General Psychol. 24, 335–354. 10.1177/1089268020961763

[B10] Golovin N. A. Vissonov R. M. (2021). On the end of the conceptual con?ict in the early theory of social systems: Sorokin PA, T. Parsons, and L. von Wiese. Sociol. J. 27, 146–163. 10.19181/socjour.2021.27.2.8091

[B11] Goodson A. (2020). Clustering by academic major at Historically Black Colleges and Universities (HBCUs). J. Negro Educ. 89:24. 10.7709/jnegroeducation.89.1.0024

[B12] Greenbaum H. Meyer L. Smith M. C. Barber A. Henderson H. Riel D.. (2016). Individual and institutional productivity in educational psychology journals from 2009 to 2014. Educ. Psychol. Rev. 28, 215–223. 10.1007/s10648-016-9360-8

[B13] Gupta A. Shukla G. Poornima S. Mohd A. Katoch J. Taneja D.. (2020). 0969 early life sleep disturbance among children with autism spectrum disorders: a questionnaire-based retrospective study. Sleep. 43(Suppl. 1), A368–A368. 10.1093/sleep/zsaa056.965

[B14] Hartson K. R. Hall L. A. Choate A. S. (2021). Stressors and resilience are associated with well-being in young adult college students. J. Am. College Health 19, 1–9. 10.1080/07448481.2021.1908309 34280317

[B15] Hossain S. Anjum A. Uddin M. E. Rahman M. A. Hossain M. F. (2019). Impacts of socio-cultural environment and lifestyle factors on the psychological health of university students in Bangladesh: a longitudinal study. J. Affect. Disord. 256:393–403. 10.1016/j.jad.2019.06.001 31226611

[B16] Hu J. Zhang H. (2021). Recognition of classroom student state features based on deep learning algorithms and machine learning. J. Intelligent Fuzzy Syst. 40, 2361–2372. 10.3233/JIFS-189232

[B17] Li C. Y. Zheng L. (2021). Analysis of tai chi ideological and political course in university based on big data and graph neural networks. Sci. Program. 9:9914908. 10.1155/2021/9914908

[B18] Li F. Guan C. (2020). The integration of socialist core values with college english teaching under the concept of “Ideological and Political Theory Teaching in All Courses”. Creative Educ. 11, 2416–2423. 10.4236/ce.2020.1111177

[B19] Lin B. (2021). Importance of ideological and political education in teaching fine arts education in higher vocational colleges. J. Contemp. Educ. Res. 5, 97–100. 10.26689/jcer.v5i5.2144

[B20] Marchingiglio R. (2021). Local institutions and public school spending under restricted suffrage: The case of post-unitary Italy. J. Econ. Behav. Organ. 188, 1351–1373. 10.1016/j.jebo.2021.06.004

[B21] Melkonian A. J. Ham L. S. Bridges A. J. Fugitt J. L. (2017). Facial emotion identification and sexual assault risk detection among college student sexual assault victims and nonvictims. J. Am. Coll. Health 65, 466–473. 10.1080/07448481.2017.1341897 28617101

[B22] Metaferia B. K. Futo J. Takacs Z. K. (2021). Parents' views on play and the goal of early childhood education in relation to children's home activity and executiv functions: a cross-cultural investigation. Front. Psychol. 12:646074. 10.3389/fpsyg.2021.646074 33981273 PMC8108989

[B23] Moody R. (2020). Contextualizing “Practice”: helping pre-service teachers unpack the ideological and sociopolitical dimensions of required practices for licensure. J. Curriculum Stud. Res. 2, 60–80. 10.46303/jcsr.2020.10

[B24] Pan H. Li Y. Zhao D. (2021). Recognizing human behaviors from surveillance videos using the SSD algorithm. J. Supercomput. 77, 6852–6870. 10.1007/s11227-020-03578-3

[B25] Qian J. Song B. Jin Z. Wang B. Chen H. (2018). Linking empowering leadership to task performance, taking charge, and voice: the mediating role of feedback-seeking. Front. Psychol. 9:25. 10.3389/fpsyg.2018.02025 30410461 PMC6209672

[B26] Renshaw T. L. Bolognino S. J. (2016). The college student subjective wellbeing questionnaire: a brief, multidimensional measure of undergraduate's covitality. J. Happiness Stud. 17, 463–484. 10.1007/s10902-014-9606-4

[B27] Reyes H. Nel R. Riquelme F. Gajardo M. Cechinel C. Mac Lean R.. (2019). Introducing low-cost sensors into the classroom settings: improving the assessment in agile practices with multimodal learning analytics. Sensors 19:3291. 10.3390/s19153291 31357476 PMC6696001

[B28] Rogoza R. Zemojtel-Piotrowska M. Kwiatkowska M. M. Kwiatkowska K. (2018). The bright, the dark, and the blue face of narcissism: the spectrum of narcissism in its relations to the metatraits of personality, self-esteem, and the nomological network of shyness, loneliness, and empathy. Front. Psychol. 9:343. 10.3389/fpsyg.2018.00343 29593627 PMC5861199

[B29] Samfira E. M. Sava F. A. (2021). Cognitive-behavioral correlates of pupil control ideology. PLoS ONE 16:e0246787. 10.1371/journal.pone.0246787 33566843 PMC7875416

[B30] Sharp L. Karadzhov D. Langan-Martin J. (2020). Delivering the first internationally accessible Massive Online Open Course (MOOC) on suicide prevention: a case study and insights into best practice. J. Perspect. Appl. Acad. Practice 8, 72–80. 10.14297/jpaap.v8i2.439

[B31] Shchepetylnykova I. Alvis S. (2020). Contribution of international development activities to comprehensive internationalization of U.S. Public Universities. J. Compar. Int. Higher Educ. 12, 15–26. 10.32674/jcihe.v12iSpring.1425

[B32] Song H. Dong H. Wang X. Tang L. (2021). Non-destructive diagnosis of grounding grids based on the electromagnetic induction impedance method. Measure. Sci. Technol. 32:115901. 10.1088/1361-6501/ac0844

[B33] Song W. Tian R. (2020). Innovation of ideological and political work in colleges and universities under new media environment relying on short video recommendation model. J. Phys. 1533, 32–34. 10.1088/1742-6596/1533/3/032034

[B34] Stoeve M. Schuldhaus D. Gamp A. Zwick C. Eskofier B. M. (2021). From the laboratory to the field: IMU-based shot andpass detection in football training and game scenarios using deep learning. Sensors 21:3071. 10.3390/s21093071 33924985 PMC8124919

[B35] Turner J. Nolen B. S. (2015). Introduction to special issue, the relevance of the situative perspective in educational psychology. Educ. Psychol. 50, 167–172. 10.1080/00461520.2015.1075404

[B36] Wang W. Young H. R. Glerum D. R. Joseph D. L. (2018). Who are the most engaged at work? A meta-analysis of personality and employee engagement. J. Organ. Behav. 39, 1330–1346. 10.1002/job.2303

[B37] Wang Y. (2020). Analysis on the construction of ideological and political education system for college students based on mobile artificial intelligence terminal. Soft Comput. 24, 8365–8375. 10.1007/s00500-020-04932-6

[B38] Wang Z. Yang L. Gao S. (2019). Pipeline magnetic flux leakage image detection algorithm based on multiscale SSD network, in IEEE Transactions on Industrial Informatics, 1–1. 10.1109/TII.2019.2926283

[B39] Wu W. Wang H. Zheng C. Wu Y. J. (2019). Effect of narcissism, psychopathy, and machiavellianism on entrepreneurial intention—the mediating of entrepreneurial self-efficacy. Front. Psychol. 10:360. 10.3389/fpsyg.2019.00360 30846958 PMC6393355

[B40] Wu Y. Song D. (2019). Gratifications for social media use in entrepreneurship courses: learners' perspective. Front. Psychol. 10:1270. 10.3389/fpsyg.2019.01270 31214081 PMC6555126

[B41] Wu Y. Wu T. Li Y. (2019). Impact of using classroom response systems on students' entrepreneurship learning experience. Comput. Human Behav. 92, 634–645. 10.1016/j.chb.2017.08.013

[B42] Xing Y. Lv C. Mo X. Hu Z. Huang C. Hang P. (2021). Toward safe and smart mobility: energy-aware deep earning for driving behavior analysis and prediction of connected vehicles, IEEE Transactions on Intelligent Transportation Systems, 1–14. 10.1109/TITS.2021.3052786

[B43] Yang J. (2021). Research on the innovation and development of ideological and political education in colleges and universities based on computer technology. J. Phys. 1744:032213. 10.1088/1742-6596/1744/3/032213

[B44] Zhang X. Sun L. (2021). Application of big data technology in the teaching model of ideological and political theory course in agricultural and forestry colleges. J. Phys. 1744:042048. 10.1088/1742-6596/1744/4/042048

